# Subjective experiences of an acceptance and mindfulness-based group intervention (Feel-Good-Group) in young people with early psychosis

**DOI:** 10.3389/fpsyt.2024.1369629

**Published:** 2024-10-07

**Authors:** Laura von Hardenberg, Karolina Leopold, Andrea Pfennig, Céline Kuhn, Michèle Kallenbach, Navid Aliakbari, Stephanie Mehl, Andreas Bechdolf

**Affiliations:** ^1^ Department of Psychiatry, Psychotherapy and Psychosomatic Medicine incorporating FRITZ and soulspace, Vivantes Hospital am Urban and Vivantes Hospital im Friedrichshain, Berlin, Germany; ^2^ Department of Psychiatry and Psychotherapy, Carl Gustav Carus University Hospital, TUD Dresden University of Technology, Dresden, Germany; ^3^ Department of Health and Social Work, University of Applied Sciences Frankfurt am Main, Frankfurt am Main, Germany; ^4^ Department of Psychiatry and Psychotherapy & Center of Mind, Brain and Behavior, Faculty of Medicine, Philipps-University Marburg, Marburg, Germany; ^5^ Department of Psychiatry and Psychotherapy, CCM, Charité Universitätsmedizin, Berlin, Germany; ^6^ German Center for Mental Health (DZPG), Charité Universitätsmedizin, Berlin, Germany

**Keywords:** mindfulness-based therapy, early psychosis, group therapy, inpatient treatment, emotion regulation, early intervention

## Abstract

**Background:**

Mindfulness-based interventions are promising psychological treatment approaches that may have more substantial long-lasting intervention effects than cognitive behavioral therapy when treating individuals with early psychosis. A pilot study analyzed mindfulness-based inpatient group therapy’s feasibility and potential efficacy (Feel-Good).

**Objective:**

This paper explores the subjective experiences of participants in the Feel-Good inpatient therapy group to gain insight into the possible changes brought about by the mindfulness-based intervention.

**Methods:**

A semi-structured change interview was used to examine the experience of ten participants who participated in the Feel-Good intervention and the additional qualitative assessment. The interviews were conducted 16 weeks after the Feel-Good group ended (16-week Follow-Up). Interviews were transcribed verbatim and analyzed using thematic analysis.

**Results:**

The analysis generated five themes—one about personal changes brought about by the Feel-Good group, three about the group therapy experience, and one about wishes/modification suggestions to change and improve the Feel-Good group. The findings suggested that the Feel-Good group was perceived as very helpful, leading to numerous changes in one’s overall well-being and relation to emotions. However, patients recommended a more directive therapeutic style and reduced time required for study assessments.

**Conclusion:**

Gathering qualitative insight from participants on the Feel-Good intervention revealed meaningful insight into patients’ experience of change processes. In addition, participant suggestions help to improve the intervention and study design to increase therapy attendance rates and treatment satisfaction, potentially increasing treatment effectiveness in the future.

**Clinical trial registration:**

ClinicalTrials.gov, identifier NCT04592042.

## 1 Introduction

Delusions and hallucinations are highly likely to transition into chronic psychotic disorders, such as schizophrenia, which are associated with detrimental consequences for people who experience such disorders, including low quality of life, diminished societal participation and functioning, poor recovery outcomes, and reduced life expectancy (1–4). Therefore, a lot of research within the past decade has focused on developing effective interventions in the early phases of psychosis (“early psychosis”: EP) to reduce the worsening of the symptoms and alleviate the burden on affected persons. Early intervention services (EIS) provide intensive, phase-specific multimodal treatment, including psychological, psychosocial, and psychopharmacological interventions, intending to decrease psychotic symptoms, improve functional and social outcomes, and reduce long-term disability (5). Findings have shown that EIS can significantly improve the outcome and reduce the negative consequences of EP (5–9).

Cognitive behavioral therapy for psychosis (CBTp) is the most frequently recommended and applied psychological intervention to date when treating psychotic disorders (10, 11) and is often used in EIS. However, the efficacy of CBTp is found to have small effect sizes in terms of reducing positive, overall, and/or negative symptoms, response to treatment, quality of life, and functioning in patients with EP and patients with chronic psychotic disorders (12–20). Also, the focus of CBTp interventions often does not cover the concerns and priorities expressed by young people with EP properly, whose primary focus lies in alleviating distressing emotions rather than psychotic symptoms (21). Patients with EP often suffer from low self-esteem, rumination, and distressing emotions (e.g., anxiety and depression) (18, 22–29), conditions that have been identified as important mediators involved in the development and maintenance of psychosis (30–32). Consequently, additional psychological interventions need to be developed that may complement CBTp approaches and prove to be more effective when targeting specific difficulties in EP while being more aligned with the treatment goals of young people with EP.

One promising psychological treatment to achieve more substantial, long-lasting intervention effects while focusing on treatment goals expressed by young people with EP (as distressing emotions) is a “third-wave” CBT approach, precisely, a mindfulness-based intervention (MBI). Mindfulness entails focusing on the present moment while observing any sensations (such as psychotic symptoms or emotional distress) in a non-judgmental and accepting way (33). Findings of two recent meta-analyses examining the effectiveness of MBIs on psychotic symptoms in randomized controlled trials, which included 43 studies (34, 35), suggest that MBIs have a small pre-post effect on reducing negative and general psychotic symptoms, re-hospitalization rates, and moderate effects on improving affective symptoms. Other meta-analyses also examining mindfulness interventions for psychosis presented small-to-moderate pre-post treatment effects on positive symptoms and improvements in symptom reduction and improved functioning that lasted over a more extended period compared to CBTp (36–40). Thus, MBIs seem promising due to their improved acceptance in young people with EP (41) and as they address difficulties in the emotional domain often reported by people with EP (e.g., enhanced distressing symptoms, maladaptive emotion regulation, limited emotional awareness; 42). However, MBIs in EP have rarely been subject to sound empirical intervention research and were seldom conducted in inpatient settings.

To address this lack of research, we conducted a pilot-controlled pre-post-trial in an inpatient setting for patients with EP to examine the feasibility of an MBI group intervention (43). The study investigated possible changes in dealing with distressing emotions and indirect improvement of psychotic symptoms by exploring the subjective experiences and attitudes of patients with EP who participated in the Feel-Good-Group intervention pilot study (8 sessions). The trial generated positive quantitative findings in reaching one’s emotional goal and emotional regulation skills, as well as a reduction in psychotic symptoms over eight weeks (post-assessment) and 16 weeks (follow-up assessment), suggesting that the Feel Good-Group intervention may be effective for improving coping strategies regarding negative emotions.

Only a few qualitative studies have investigated how patients with EP experience MBIs (41, 44–48), most of which were feasibility pilot studies. The number of participants included in the studies ranged from 9-19 patients. Findings suggest that patients with EP reported an increased sense of self-understanding and acceptance (41), better coping with stress and rumination (46), and fewer mood swings (44). These findings suggest that participants with EP benefit from using mindfulness. However, all qualitative studies were performed in outpatient settings when the psychotic symptoms of the patients were not as acute anymore. Furthermore, these studies did not focus on emotion regulation. A review examining the clinical effects of MBIs in patients with early psychosis highlighted the fact that emotion regulation has been poorly assessed to date (49). Thus, using a qualitative design, this study explored participants’ subjective experiences regarding changes in dealing with distressing emotions with the Feel-Good-Group intervention in an inpatient setting.

## 2 Methods

### 2.1 Study design and setting

This analysis was conducted within the qualitative section of the Feel-Good feasibility pilot trial (‘Feasibility and Efficacy of an Acceptance and Mindfulness-Based Group Intervention for Young People with Early Psychosis’; 43), which was approved by the Ethics Committee of the Psychologische Hochschule Berlin and registered at ClinicalTrials.gov (Identifier: NCT 02787122). Participants were recruited between November 2020 and November 2021 from the specialized inpatient and day-treatment ward “Frühinterventions- und Therapiezentrum; FRITZ” (early intervention and therapy center) in Berlin. All patients who participated in the Feel-Good trial were asked at the 16-week post-assessment whether they were interested in an additional qualitative assessment. They or their legal guardian gave written informed consent.

### 2.2 Participants

**Figure 1:**
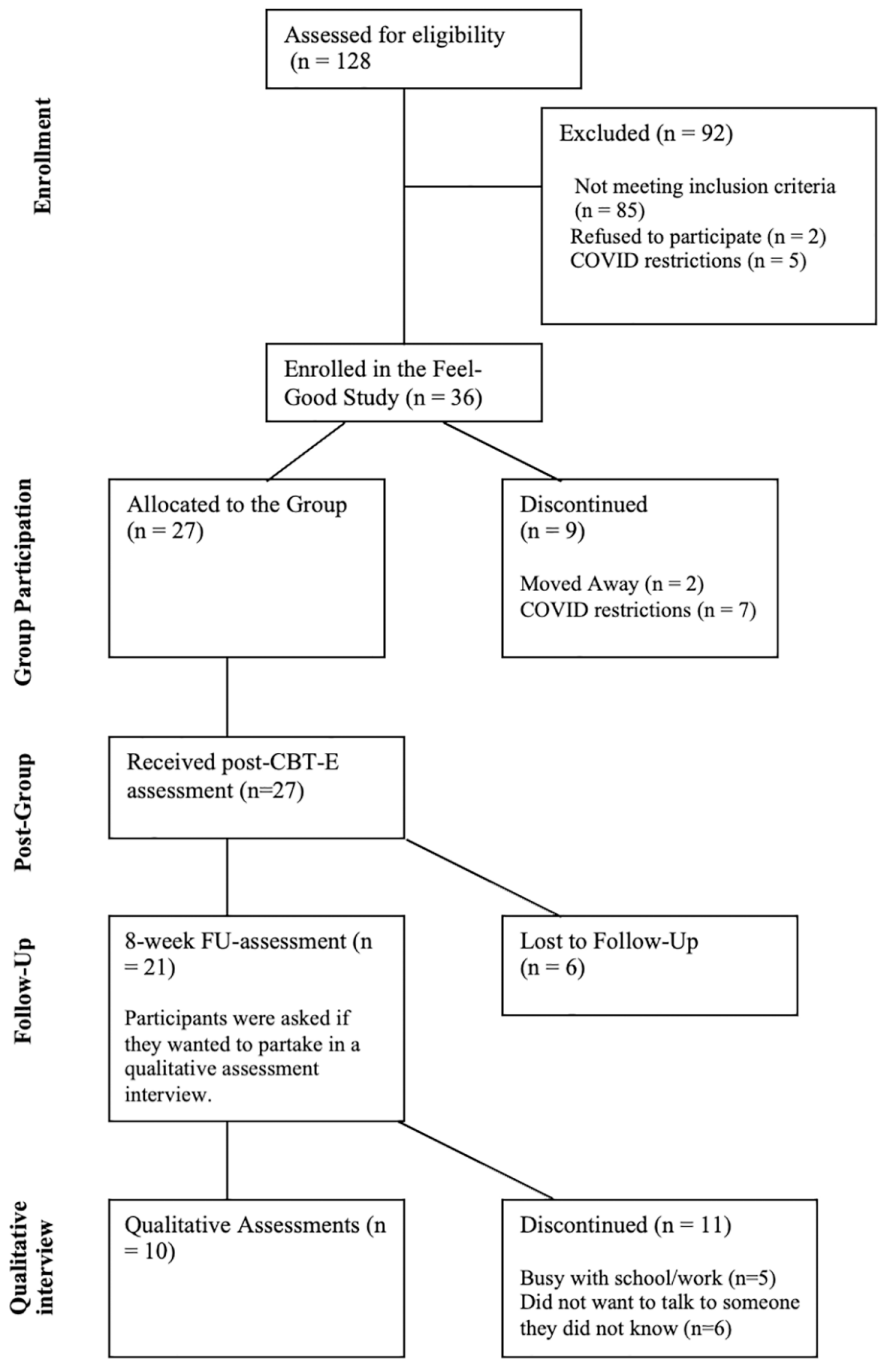
Flowchart of the Feel-Good Study.

Participants were eligible to participate in the present add-on study if they met the following inclusion criteria: i) age between 17 and 65 years, ii) diagnosis of schizophrenia, schizoaffective disorder, psychotic disorder, or bipolar disorder with psychotic symptoms using the ICD-10, iii) onset of the first psychotic episode or first presentation to mental health services in the last five years, iv) estimated verbal intelligence score of ≥ 80 in the German Mehrfachwahl-Wortschatz-Intelligenztest (MWT-B; 50), v) absence of current suicidal tendencies, vi) no diagnosis of dementia, and vii) proficient use and comprehension of the German language and vii) if they endorsed participation in the additional qualitative assessment (for details, see 43). After giving written consent, all participating patients (*n* = 21) were invited by a research assistant affiliated with an independent research institute (CK) to participate in an additional qualitative assessment interview on their perception of change and feasibility of the Feel-Good group (see **Figure 1**).

### 2.3 Intervention

#### 2.3.1 Feel-Good Group intervention

**Figure 2:**
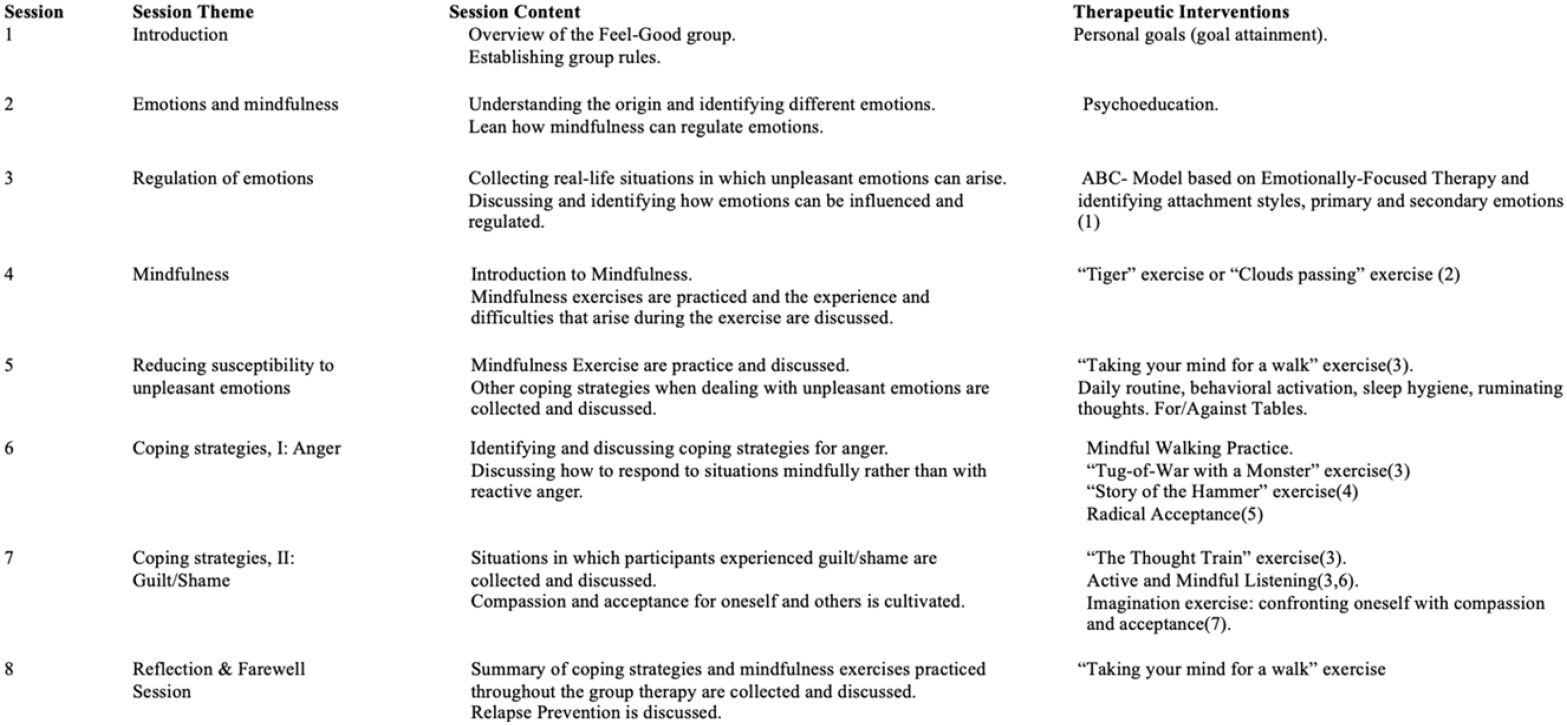
Feel-Good Therapy Session Overview. 1. Greenberg LS, Goldman RN. Clinical Handbook of Emotion-Focused Therapy. Ist edition. Wasington, DC: American Psychological Association; 2018.534 p. 2. Wells A. Metacognitive Therapy for Anxiety and Depression. Reprint edition. New York, NY: Guilford Publications; 2011. 316 p. 3. Hayes SC, Strosahl KD, Wilson KG. Acceptance and Commitment Therapy, Second Edition: The Process and Practice of Mindful Change. Guilford Press; 2011. 417 p. 4. Watzlawick P. The situation is Hopeless, But Not Serious: The Pursuit of Unhappiness. Reprint edition. New York: Norton and Company; 1993.128 p. 5. Robins CJ, Schmidt III H, Linehan MM. Dialectical Behavior Therapy: Synthesizing Radical Acceptance with Skillful Means. In: Mindfulness and acceptance: Expanding the cognitive-behavioral tradition. New York, NY, US: The Guillford Press; 2004. p. 30-44. 6. Greenberg LS, Klosterziel R. Emotionsfokussierte Therapie. Berlin: Reinhard; 2011. 7. Gilbert P. Compassion-Focused Therapy: Distinctive Features. London: Routledge Chapman & Hall; 2010.

The “Feel-Good” Group was an open-enrolling intervention consisting of 8 therapy sessions (50 minutes) offered twice weekly for 6-8 patients over one month in addition to treatment as usual on the FRITZ ward (pharmacology, individual and group psychotherapy, and socio-therapeutic approaches) (51). Patients could join the group therapy sessions anytime and participate in 8 consequent sessions. The Feel-Good group consisted of a combination of classic CBT interventions, such as psychoeducation, and elements from numerous third-wave CBT approaches, including Acceptance and Commitment Therapy (52), Emotion-Focused Therapy (53), Compassion-Focused Therapy (54), and Schema Therapy (55, 56). For a detailed overview of the individual sessions and the manual, see **Figure 2** and Mehl et al. (57). Participants had to attend at least six out of eight sessions to be included in the study analyses.

#### 2.3.2 Measures

##### 2.3.2.1 Semi-structured interview

The qualitative assessment was conducted in a semi-structured format using an abbreviated and adapted version of the Client Change Interview (58). The interview was conducted using open-ended questions that included suggested prompts to explore participants’ experiences in the Feel-Good Group (1): any personal changes since the start of the therapy; (2) overall experience within the group; (3) helpful aspects of the therapy; (4) distressing aspects of the therapy; and (5) wishes and suggestions to change/modify the therapy. Interviews were conducted over the phone or via Zoom (only audio) and were audio-recorded. Interviews were conducted in German. The quotations included in this paper were translated verbatim from German to English by a native speaker (LH). To protect the anonymity of the participants, the pronouns them/they will be used.

##### 2.3.2.2 Demographical and clinical symptoms

Numerous measures (interviews and questionnaires) were used to compare differences in clinical symptoms at baseline, 8 weeks post-intervention, and 16 weeks follow-up (for a detailed overview and descriptions of all measures used, see **Supplementary Table 1**). The time taken for study assessments differed between patients, with numerous factors having an influence, such as side effects of medications (i.e., drowsiness), cognitive deficits due to the illness, and/or a comorbid diagnosis. Two patients were not able to concentrate for extended periods of time at baseline and, therefore, asked for assistance in filling out the self-questionnaires. Screening and baseline measures were conducted over two weeks and split into several sessions. The length of the assessment sessions depended on how participants felt and the amount of information they revealed. In total, screening and assessment measures at baseline ranged between 265 and 520 minutes.

#### 2.3.3 Therapist and raters

The therapists in this study consisted of two clinical psychologists (M.Sc.) enrolled in their final year of German postgraduate training (5 years) to become certified CBT therapists. Both therapists have worked on the FRITZ ward for at least three years and underwent additional training (8h) on the Feel-Good intervention. One of the study PIs (SM) supervised both therapists monthly. An independent psychologist (M.Sc.) with extensive research history and experience with the utilized interviews and questionnaires conducted the study assessments and ratings. An additional psychologist (B.Sc.) who does not work on the FRITZ ward and has no prior contact with the study participants conducted the qualitative interviews.

### 2.4 Analysis

#### 2.4.1 Qualitative analysis

Each interview was recorded and transcribed using the MAXQDA program (59). Transcription, coding, and identification of themes was conducted by CK, who was not involved in providing treatment nor affiliated with the hospital. Following the Braun and Clarke stages, an inductive thematic analysis (TA) was conducted (60). Each interview was read several times, and data extracts were coded systematically. This means that keywords or phrases that stood out in the interview and pertained to one of the five main themes explored through the semi-structured interview were identified. Codes were then reviewed for patterns and assigned to superordinate emerging themes by both CK and LH. All emerging subthemes were then reviewed at the level of coded data extracts to ensure a coherent pattern was formed. If data extracts did not fit into the subthemes, new ones were developed, or existing ones were reviewed to accommodate extracts. The generated subthemes were then examined to assess whether they reflected the dataset and did not merely represent a singular comment reported by one participant. If they did represent a singular comment reported, they were discarded (i.e., therapists’ competence, personal motivation, additional emotions to be discussed, such as anxiety or sadness). The principal investigators, AB and SM, conducted the final stage of deciding upon and refining the final themes. The consensus on themes proved to be high from the beginning.

#### 2.4.2 Quantitative analysis

Independent t-tests for continuous data and chi-square tests for categorical data were conducted to obtain insight into whether participants who participated in the interview differed from those who did not. Quantitative analyses were two-sided tests with an alpha error of 5% conducted using SPSS (61). Of note is the small sample size used to compare the two groups. The analysis will provide an idea of whether group differences may exist, but no exact conclusions can be drawn from the results.

## 3 Results

Ten out of twenty-one participants (47.62%) at follow-up accepted the invitation to partake in the qualitative assessment interview, gave verbal and written informed consent, and were interviewed. For sociodemographic and clinical characteristics, see **Table 1**. Of the eleven participants who did not want to partake in the qualitative interview, five reported being busy with work or university, and six reported not wanting to talk to someone they did not know. No significant differences in sociodemographic or clinical characteristics between participants who accepted or declined the invite for the interview were found (see **Supplementary Table 2**).

**Table 1:** Sociodemographic and clinical characteristics of participants at baseline.

Sociodemographic Baseline characteristics	Sample *(n=10)*		Clinical Baseline Characteristics	Sample *(n=10)*	
	M/N	SD/%/(Range)		M/N	SD/%/(Range)
Age (years)	24.2	6.8 (17-39)	Primary Diagnosis^b^:		
Gender (Female)	4	40%	295.9	5	50%
Education (years)	13.4	2.5 (10-18.5)	296.55	1	10%
Nationality:			292.9^c^		
German	8	90%	Cannabis	1	10%
Turkish	1	10%	Hallucinogens	1	10%
Other	1	10%	Multiple Drug Use	2	20%
Number of Psychiatric Episodes:			DUP (days)	299.2	492.2 (4-1460)
1 Episode	5	50%	Total PANSS score	70.8	20.1 (43-96)
2 Episodes	3	30%	Current Psychotropic Medications:	6	12
3+ Episodes	2	20%	AP	10	100%
			MS	1	10%

M, Mean; N, Number; SD, Standard deviation; DUP, Duration of Untreated Psychosis; PANSS, Positive and Negative Syndrome Scale Total Score; AP, Antipsychotics; MS, Mood Stabilizers.

b DSM-V Codes Reported.

c Drug-induced psychotic disorder was diagnosed at this time point, as psychotic symptoms occurred solely while consuming drugs. Diagnosis may change throughout the course of the illness.

The qualitative data analysis generated five themes: one relating to aspects of personal change, three relating to the effectiveness and experience of the group therapy, and one relating to wishes/modifications of the intervention. Themes one to four and associated subthemes will be described below and will entail participant quotes to illustrate the theme content. Due to the large number of themes collected, patient quotations are tabulated. Theme five (wishes and suggestions to change/modify the therapy) is given in the supplements (see Text S2).

### 3.1 Theme 1: personal changes

Participants were asked to describe any changes they noticed and provide possible explanations. Half of the participants (*n*=5) denied noticing any changes when answering directly; however, they mentioned personal changes they attributed to the Feel-Good group throughout the interview. The changes reflected an improvement in (1) well-being, (2) understanding and communicating about emotions, and (3) coping with emotions.

#### 3.1.1 Well-being

Most participants (8 out of 10) reported an overall improvement in their well-being due to the therapy. There were individual differences in terms of how well-being was defined. For P10, the group therapy gave them the feeling of being stronger, whereas for P3 and P9, the treatment helped them become calmer (see **Table 2**). The changes through the Feel-Good treatment for P3 were also noticed by friends. For other participants, greater well-being was represented by being in a better mood. While some participants described a “positive” (P4) or “fresh” (P8) mood, others described feeling “less sad” (P2) or noticing improvements in their daily activities (“getting out of bed”) to reduce negative feelings (P7). Furthermore, some participants also described being more “open toward new things” (P4, P6), which continued to be present beyond the Feel-Good group and has improved their social life and feeling more connected with others.

**Table 2:** Patient quotations regarding any changes they had noticed in themselves and possible explanations for these changes.

Participant	Quotation
Wellbeing	
P10	“My concluding opinion is that the Feel-Good Group […] helped me find overall stability in my life and with fighting against the side effects of the psychosis.”
P3	“I did not have that much patience and after [the Feel-Good Group], a lot of my friends told me that I have gotten much calmer.”
P9	“Well, I did get a bit calmer, even though I already am a relatively calm person, but yeah, maybe more thoughtful as well.”
Understanding and communicating about emotions	
P5	“Because we talked a lot about feelings and emotions, I definitely understand a few feelings much better. […] That way I got better, I would say, at understanding my own body when it sends me specific signals.”
P10	“During the last time I took a lot of medications and did not have that many emotions. But now they are starting to come back, and I think it’s good to have talked about them, on how to deal with them.”
Coping with emotions	
P1	“I try to observe when tense phases occur to deal with them better. That was the main thing that stayed with me. […] In principle by being more consciously aware of my own thought processes, my own feelings, etc.”
P6	“And in some situations, I cope differently with my emotions. […] Or with other emotions like Anger […] I cope better, by not letting it out on others. Maybe it is better to first withdraw and let the anger out by screaming into a pillow or so before you let it out on others. That you try to deal with it by yourself first. And then, after you thought about it, you can talk to other people about it […]“
P8	“I can now sit down and say ‘Okay, I am currently angry. Something silly got to me.’ […] I have this anger and I deal with it and redirect it into a happier path or in a good, positive path and for me that is something amazing. “

#### 3.1.2 Understanding and communicating about emotions

Half of the participants (*n*=5) reported an improved understanding of their emotions. Being able to take the time to “identify” and understand “how emotions come about” (P9) has helped them gain a more in-depth understanding of themselves. This was further substantiated by P5, who also became more aware of mental and bodily needs by learning about emotions (see **Table 2**). Furthermore, a better understanding of emotions and improving communication within the Feel-Good Group helped P6 become “more open” and “more willing” to talk to other people again. For P10, the therapy was “helpful” as understanding their own emotions also helped them better understand their actions and reactions toward external stimuli (see **Table 2**)

#### 3.1.3 Coping with emotions

Half of the patients (*n*=5) reported that the Feel-Good Group helped them to improve their coping skills regarding their emotions. P1 spoke on how the group helped them pay more attention to anxious moments, which in turn allowed them to utilize the learned strategies in the group to help cope and regulate their anxiety (see **Table 2**). Furthermore, the group also helped P6 take their time to observe and process their emotions without immediately reacting in dysfunctional ways (see **Table 2**). Also, P8 reported feeling more secure about confronting personal emotions instead of trying to “repress them,” as they learned numerous strategies in the Feel-Good Group to cope with distressing emotions (see **Table 2**). Some patients (*n*=3) reported that mindfulness, specifically the concept of “radical acceptance” helped in terms of coping with distressing emotions, specifically for anxiety (P1, P2) and for “ruminating and spiraling” thoughts (P10).

### 3.2 Theme 2: overall experience

#### 3.2.1 Positive experiences

Most participants (*n*=8) gave positive feedback on their experiences within the group. The feedback ranged from “I thought the Feel-Good Group was very good” (P10) to “Overall, I thought it was quite good” (P2) to “I am very satisfied with the Feel-Good Group” (P4). There were numerous factors associated with a positive experience. One main factor contributing to an overall positive experience within the group was the topic ‘emotions’ as mentioned by P5 and P6 (see **Table 3**). Furthermore, the frequency of the group sessions contributed to the positive experience within the group. P10 even expressed looking forward to the group sessions (see **Table 3**). Lastly, participants reflected that receiving the intervention in a group setting benefited them. They were happy “not to have been alone” (P9) and thought the group therapists and other participants were “very nice” (P6).

**Table 3:** Patient quotations regarding their overall experience in the Feel-Good group.

Participant	Quotation
Positive experiences	
P5	“[…] and I found [the Group] actually very interesting because we talked about the topic emotions […] quite intensively.”
P6	“[…] and the topics we discussed I found very good overall.”
P10	“I thought it was nice to go there twice a week to the session, I always looked forward to it.”
Negative experiences	
P1	“At the beginning it was a bit tough. But I have to say, that was also because of the other participants. Because they were more hesitant to talk about their problems.”
P9	“It usually lasted an hour and then I found it to be a bit exhausting. To sit on the chair. You had to listen a lot. There was a lot of talking. A bit of perseverance was required. But I just participated. “

#### 3.2.2 Negative experiences

Two participants spoke about negative experiences they encountered in the Feel-Good Group. For P1, it was frustrating that sometimes other participants did not actively participate in the group (see **Table 3**). For P9, the duration, accommodation, and the group setting were sometimes perceived as exhausting (see **Table 3**).

#### 3.2.3 Ambivalent experience

One participant was unable to form a decisive answer on their experience within the group. The question on the overall experience was answered using mutually exclusive words “good” and “not good” (P8).

#### 3.2.4 Overall effectiveness

All participants agreed that the group, despite the positive or negative experiences, was “helpful” (P1, P2, P3, P4, P6, P7, P8, P9). For P5 and P10, the group was deemed “very helpful”. When asked whether the participants would recommend the group to friends, all participants responded with “yes”. P2 revealed to have already recommended the group to two friends.

### 3.3 Theme 3: helpful aspects

In addition to noticing changes in themselves, participants were asked to identify specific aspects of the Feel-Good group that were the most helpful.

#### 3.3.1 Emotion regulation

Emotion regulation was the most mentioned aspect and, thus, probably the most helpful of the interventions learned in the Feel-Good group (*n*=8). Participants P3 and P4 explained how the Feel-Good group helped them explore their negative emotions and re-direct their energy into having a more positive outlook on situations (See **Table 4**). Also, P7 revealed achieving changes in the intensity of emotions by utilizing strategies learned in the Feel-Good group (see **Table 4**). For P2, specifically, breathing techniques were able to reduce anxiety and help “center” them. This was further substantiated by P5, who also used breathing techniques to help reduce stress. Lastly, participants also talked about how strategies learned in the Feel-Good group helped decrease the amount of time negative emotions were present. For P1, “radical acceptance” helped to avoid “uncomfortable situations in which I would quickly get angry”. Similar experiences were reported by P8 (see **Table 4**). Furthermore, P10 reported to have spent less time with “ruminating and spiraling thoughts” due to using mindfulness techniques instead.

**Table 4:** Patient quotations regarding aspects of the Feel-Good group that were deemed helpful.

Participant	Quotation
Emotion regulation	
P3	“And when a negative emotion comes, one must explore where it comes from. And to find the reason, […] and always try to find a solution, so to always ask questions and look for an answer.”
P4	“[…] I think that if you search for [reasons why negative emotions are there] you then only perceive things are not so good and you can change it to something positive. That you then perceive it as positive.”
P7	“And yeah, […], well the exercise regarding negative emptions, what you can do so that they will become a bit less.”
P8	“So, I don’t immediately freak out when I feel a certain way, rather I sit down, observe it for a bit, deal with the emotion and then assess how I want to proceed.”
Education	
P10	“[…] what are emotions. That was the most important thing. I never dealt with that before. [The emotions] were just somehow always there, or then suddenly it was no longer there, or it was very intense because of the illness. […] That I found helpful, that it is important and relevant and to understand what it does to someone.”
P6	“So, I definitely found it helpful in the sense that, I learnt how to talk about my feelings and emotions.”
P1	“In principle, I can respond better or more effectively to the feelings I have or am able to sort them better.”
P9	“Helpful, well, yes. […] I think, yes, we talked about feelings, and it was explained why feelings arise, and yes, I thought that was good, it was interesting to analyze it like that.”
P5	“We talked about, partially, what purpose certain feelings have and how they always want to communicate something to you, so I was able to understand my body better, I would say, when it wanted to give me certain signals.”
P3	“That we talked about some feelings, for example, shame, how you act and how you feel and I learned a few things from [the Feel-Good group], like that you sweat and then you blush when you’re ashamed and definitely, there were a few important things that we learnt. “
P7	“I found it helpful, I would say, that it was explained, for example with fear […] that the feeling has a reason, why you feel fear and that its not just bad […]. “
Exchange and support	
P6	“That you can definitely talk openly with each other in the group and that everyone takes part in it somehow and that you build up trust, so that you know, that you can speak about everything in the group and that outside of the group people won’t speak about it.”
P8	“So, we brought up different situations on the topic of feelings and worked in-depth on them. Until we, well, we worked in a solution-oriented manner, and I thought that was pretty good. “
P10	“So, I found it very helpful that we talked about concrete cases […] Then another participant always contributed something of their own, and I always thought that was good to somehow talk about it again, to go through the situation again and then to discuss it with the other participants.”
Talking about one’s problem	
P2	“And we also talked a lot about fear. And I was able to share a few of my own stories and experiences. And that just felt really good.”
P5	“I found it very pleasant to talk about it too, rather than to deal with it myself as per usual.”
P6	“So, I also learned a lot just by talking about feelings or learning something new about feelings or emotions. And it was definitely new for me to even talk about it. And that’s why it already helped me immensely. “
P5	“I would say that you talked to different people, and also allowed yourself to say a little bit more intimate things, for example if you had problems with something, that you allow others to listen to you and they can give you tips […].”
Connectedness with others	
P2	“But it was also good to get a different perspective and to realize that maybe you’re not really alone with some things. “
P9	“Because you don’t feel quite so alone. And you also got to know your fellow patients a bit.”
P10	“And yeah, […], you come together with others. I’m very introverted, so getting together with other people who have similar illnesses and to see, how they handle it and learn how to deal with it together. “
P6	“And the group definitely made me feel understood. Even in the group where others weren’t feeling well and others who were struggling with similar problems to re-integrate into everyday life, in that sense already helped me. That you are working together with people, who feel similar to you. And that it definitely does get better, that it will be the way it was before. “
P5	“And that you can talk about it. That is a really big aspect, that you can talk about how you feel, and so when you feel really bad that you maybe then receive help.”
Mindfulness	
P8	“So, first of all I wanted to go on the fantasy trip, and you had to observe and see how it affects you. And then I took on a bit more of an observer role or a listening role. And what makes [the trip] so valuable? It was creative, had a relaxing effect on me and was just great, yes.”
P1	“The mindfulness exercises were basically a good idea and were implemented well.”

#### 3.3.2 Education

Most participants (*n*=8) also found the educative aspect of the Feel-Good group helpful. One participant reported never talking about emotions in such depth and how talking about emotions already helped. This was substantiated by P6 and P10, who noted how it helped develop how they communicate about emotions. Furthermore, understanding the function of emotions and learning about the evolution of emotions was reported as helpful by P1 and P9 (see **Table 4**). Some participants (P5 and P3) reported how they became more aware of their bodily signals after learning how emotions can be expressed (see **Table 4**). Additionally, it was also found helpful to know that there are positive aspects of emotions that are usually deemed “negative”, such as fear, as described by P7 (see **Table 4**).

#### 3.3.3 Exchange and support

Participants (*n*=8) found exchanging information and experiences with and receiving support from others in the group helpful. The group climate was referred to as very “open” by P6 and P2, which helped them talk more about themselves. For P6, this made them less wary of addressing topics within the group (see **Table 4**). Some participants (P8 and P10) also spoke about how communicating about one’s own experiences, receiving feedback, and finding solutions with other group members to cope with emotions in those situations was helpful.

#### 3.3.4 Talking about one’s problem

Some participants (*n*=6) found talking about their problems in the group very helpful. Some found the experience of opening up and sharing their experiences beneficial (see **Table 4** for descriptions by P2, P5, and P6). Other participants (P5) spoke about how it was helpful to talk about one problem because of the feedback and responses from the other group members (see **Table 4**).

#### 3.3.5 Connectedness with others

Half of the participants (*n*=5) considered the relationship with other people in similar situations to be helpful. Seeing other people struggle with similar problems made some participants feel less lonely and isolated (P2, P9, and P10; see **Table 4**). Furthermore, talking about emotions allowed participants to realize that other people struggle with similar emotional problems while also allowing room for hope that the situations can change and improve (see P6, **Table 4**). For P5, connectedness to other people was achieved when talking about how bad they felt and receiving help (see **Table 4**).

#### 3.3.6 Mindfulness

Numerous participants (*n*=6) specifically mentioned mindfulness exercises as helpful. The learned strategies, breathing techniques, and/or fantasy trips were considered good ideas, well implemented, and perceived as valuable input (see descriptions of P8 and P1 in **Table 4**).

### 3.4 Theme 4: distressing aspects

Participants were also asked to identify distressing, difficult, inhibiting, or even missing aspects of the Feel-Good group. Overall, few participants reported distressing aspects. The relation between helpful and distressing aspects was 4:1 (278 vs. 64 coded text passages).

#### 3.4.1 Personal information

Two participants (P2 and P6) spoke about the difficulty of opening up and revealing personal information to strangers during the first sessions of the Feel-Good group (see **Table 5**). One participant (P6) also expressed inhibition to share personal information as the sessions were audio-recorded (see **Table 5**). Two participants (P10 and P8) reported difficulties in supporting other group members at times. Meanwhile, for P10, there were strong feelings of compassion and regret for fellow patients who shared their experiences. For P8, it was not finding words or providing a good answer or helpful solutions that were perceived as difficult (see **Table 5**).

**Table 5:** Patient quotations regarding aspects of the Feel-Good group that were deemed distressing.

Participant	Quotation
Personal information	
P2	“So, of course it is not so easy, to open up in front of strangers, because I mean, I knew a few people in the group, but a few I did not know.”
P6	“Yes, it was difficult when you had to share a personal example of yourself. Not to think about: “Well, how might the others think if I talk about it?”, but rather to just reveal it openly and to know that the others can only help you.”
P6	“What was perhaps a bit difficult was that everything was recorded and, of course, kept under wraps. And yet somehow, at the beginning when I wasn’t so open, it gave me a bit of inhibition to partake in the group, because it might be recorded, and someone could say the wrong thing.”
P10	“When other people say that they are afraid of being followed on the train, then of course you have a little bit of compassion and a little bit of regret. But then you try to make [these feelings] go away by providing advice and encouragements. “
P8	“Yes, difficult when I didn’t have an answer.”
Participation engagement	
P2	“It was a bit stiff. But that was also because of the participants, I have to say. Because some of them weren’t as open to talk about their things.”
P1	“Sometimes there was little conversation. Somehow because not that many people wanted to say anything. “
P10	“I would almost rather do the group now. Now that I’m feeling better than back then because I was heavily sedated because of the medication and sometimes fell asleep during the group. […]. I noticed that with the others as well, that they were heavily [sedated].”
Implementation into everyday life	
P3	“It’s probably still difficult for me. “
P10	“Yes, you fall back into your old pattern/habits relatively quickly. I think that was more a problem that you somehow after the hospital stay think: ‘Man, somehow everything is getting better and now you have learnt a lot.’ But then your quickly slip back into your old self. That is what makes it the hardest to implement what you have learnt. “
P4	“[…] dealing with it myself and doing everything myself, I think that’s what’s still difficult for me […]“
P1	“Ultimately, it depends on you doing these mindfulness exercises yourself. Maybe one could have delved a little more into the topic of how you can […] motivate yourself to actually do the exercises more often so that you get a better feel for them.”
Study design	
P4	“P4: With some of the exercises I did not cope well with, but I do not know why, maybe because I did not cope so well. I: Okay, what exercises were there that you did not cope well with? P4: I think it was the one with the tiger that did not affect me. “
P5	“Once I had, we had, to watch and observe a tiger. I found that a bit difficult and I also mentioned that in the group. But we also had a different method, and that was to simply watch trains go by at a train station, and since that’s much more like everyday life instead of a tiger, because you don’t really see a tiger. “
P2	“Maybe even more suggestions for solutions from the therapists. Because there was a lot of exchange and everyone shared their experiences, but they aren’t really experts. So maybe a little more guidance.”
P9	“The only negative thing was that it was sometimes so inactive that you just sat and listened and talked a lot […], that over time I sometimes got a little tired from all the sitting.”
P10	“During the study discussions you always had to talk a lot about your illness, which was quite churning and then somehow you were left alone with it. And when you’re already in a sensitive phase, […], I found it relatively harsh […].”

#### 3.4.2 Participant engagement

Two participants (P2 and P1) were unhappy regarding the amount of active participation of other members in the group. There was a lack of willingness to talk, no individual examples were presented, and general scenarios had to be devised to discuss possible solutions when dealing with emotions (see **Table 5**). One participant (P10) reported difficulties participating in the Feel-Good group with other participants because of the potential side effects of the medication (sedation) (see **Table 5**).

#### 3.4.3 Implementation into everyday life

Some participants (*n*=4) reported difficulties implementing the learned strategies and exercises into their everyday lives (see P3 and P10 in **Table 5**). Two participants (P4 and P1) felt overwhelmed with the exercises when left to their own devices, with one participant expressing the desire for more guidance to motivate himself in performing the exercises (see **Table 5**).

#### 3.4.4 Study design

Some singular statements were made about the study design and setting aspects. These ranged from criticisms about individual exercises used in the study to the design of the sessions and the data acquisition (clinical data) that took place on top of the group therapy.

Two participants (P4 and P5) reported difficulties with a specific mindfulness exercise (“Tiger Task”) that made it more difficult for them to notice the effects mindfulness can have. Using other exercises that are more relevant to everyday life and easier to visualize made it easier to use mindfulness strategies (see **Table 5**). A further criticism reported by P5 was the lack of examples presented in the sessions to practice finding solutions in terms of emotion regulation. This was substantiated by P2, who noted some lack of expert knowledge and that they would have benefitted more if there had been more solution-oriented input from the therapists (see **Table 5**).

One participant (P9) reported difficulties with the length of the group sessions and the need for more interaction within them (see **Table 5**). Another participant (P10) criticized the sessions in which clinical data was acquired. They found the assessments, specifically the baseline assessment, very long and intense, and discussing all the clinical symptoms in detail was upsetting. They did not feel supported or cared for after these assessments (see **Table 5**).

## 4 Discussion

The quantitative findings from the pilot study suggest that the Feel-Good group may be an effective intervention for improving emotion regulation in patients with EP and thus indirectly improving overall psychotic symptoms. However, more understanding of the changes related to the Feel-Good group was needed and how these changes may have led to overall improvement in clinical symptoms. The presented analysis offers insight into how and why patients’ overall improvements in psychotic symptoms may be changing during the Feel-Good group.

Participants reported that the group therapy initiated several personal changes. There were improvements in (1) overall well-being, (2) gaining a comprehensive understanding of emotions, and (3) improving coping strategies addressing distressing emotions. These findings suggest that understanding and using adaptive strategies for efficient emotion regulation may improve overall well-being. This may be because patients were able to cope better with their distressing emotions, which in turn led to an increase in self-efficacy. As a result, realizing one’s agency over distressing emotions may lead to feeling stronger and coping better with situations. This is substantiated by findings from other qualitative studies, showing that participants with EP in outpatient settings coped better with stress and felt more in control over situations (41, 45, 46). In addition, the adaptive strategies learned in the Feel-Good group may have reduced maladaptive strategies, such as rumination, that frequently occur in patients with EP (62). This supports findings in participants with EP in outpatient settings, who reported less rumination and procrastination tendencies (46).

Of note is the finding that there was a high acceptance of the group, as most participants (*n*=8) gave positive feedback on the overall group therapy experience. Participants considered the Feel-Good group helpful and recommended group therapy to friends. Furthermore, more helpful aspects were identified and mentioned in comparison to distressing aspects (ratio 4:1). A possible explanation could be that participants did not want to upset the research team. However, we tried to minimize this effect by having a research assistant affiliated with an independent research institute (CK) conduct the interviews. Also, patients who agreed to the interview may have been more therapy-motivated than other participants, which may be why more helpful aspects were mentioned. However, drop-outs did not occur throughout the intervention, only between post-group and follow-up (16 weeks), suggesting that most participants had a positive perception of the Feel-Good group.

Participants revealed how psychoeducation on emotions and improvements in emotion regulation through mindfulness were the most helpful aspects of the Feel-Good intervention. These findings suggest that the information conveyed in the group was well understood and that strategies could be implemented and practiced in the group. Identifying mindfulness as a critical component to help relax and distract was also substantiated by other qualitative findings with patients with EP in outpatient settings (44, 47). Patients also reported interactional aspects as helpful (connecting with others, speaking about one’s problem, exchanging and supporting in the group). Research has shown that individuals with EP experience high levels of loneliness, poor perceived social support, and reduced social networks (63). Thus, during group therapy, individuals may feel less isolated once they realize others have similar problems, leading to destigmatization, lessening the burden of the illness and associated consequences, and increasing one’s well-being. Other research with EP in outpatient settings also found interaction among the group reported as helpful by participants (47).

Two distressing subthemes (patient engagement and revealing personal information), often interrelated, are common occurrences at the beginning of individual or group therapies (64, 65) and have also been reported by another study (47). Furthermore, as this was an open-enrollment group, there was always a period of adaptation and flexibility necessary for group cohesion to build (66). The other two distressing subthemes referred to the study design and difficulties implementing taught strategies into everyday life. Changes and modifications to improve these themes will be further discussed under ‘implications for future research.’

### 4.1 Strengths and limitations

As this analysis is the first to evaluate the Feel-Good intervention qualitatively, some limitations must be addressed. First, it was part of a pilot project examining the feasibility of the Feel-Good group and thus was not a pre-planned qualitative study exploring participants’ experience with the intervention. Second, even though no significant differences were found between the sample who participated in the interview and participants who declined the invitation, the small sample size limits the validity of the data. Therefore, no attempts can be made to generalize these findings. Another limitation is that there was no control group. Thus, it is not possible to attribute the improvements participants had solely to the Feel-Good group. Instead, the improvements witnessed may result from the Feel-Good Group and other therapies (including medication) provided throughout their inpatient stay or remission of psychosis. Strengths of this study include the focus on the reports of service users and not professionals that the interviews were conducted by a research assistant not ‘actively’ involved in the study, the interviews were conducted online, which aided in the feeling of anonymity, and the semi-structured nature of the interview allowing for participants to delve into themes/topics they value or find important.

Furthermore, this analysis, in combination with the quantitative study, provides a more in-depth and comprehensive glimpse into the experience participants had with the Feel-Good intervention and identifies specific factors resulting in positive changes. Over the last decade, there has been a push to use quantitative and qualitative research methods to assess and evaluate therapeutic interventions (67, 68). The combination of both research methods may inform us to improve interventions and study designs that help reduce therapy drop-out rates, increase treatment satisfaction, and thus impact future treatment-seeking behavior (69, 70). Also, it may help to identify factors that should be included when designing and delivering interventions, as quantitative studies alone do not fully capture participant’s views on the therapy (69, 71).

### 4.2 Implications for future research

Participants in this analysis mentioned the desire for expert knowledge to have been more present in the Feel-Good group. This finding might suggest that patients prefer a less Socratic therapeutic style, leading the group to form solutions based on Socratic questions but a more directive therapeutic style, presenting more examples and case vignettes. As the topic of coping with distressing emotions tends to be an abstract topic, it might be helpful to improve the Feel-Good intervention manual in advising therapists to adapt a more directive therapeutic style, using more examples and case vignettes, especially if patients are in an early phase of antipsychotic medication and experience sedation. In addition, to the best of our knowledge, there is only little research available on preferred therapeutic styles for individual and group interventions for patients with psychosis. Exploring this question further in future experimental and intervention studies would be interesting.

In addition, including a peer support worker (PSW) should be considered when designing the study. PSWs have experienced their own mental health issues and went through a structured training program to help and support other’s recovery from mental health conditions (72). Thus, their input when designing therapy studies is vital, as they have first-hand experience with mental health conditions and can articulate possible ramifications regarding the study design, setting, specific exercises, educational aspects, etc. They may, for example, have identified that participants may face difficulties with the tiger exercise and suggested using a different exercise that the participants would have better received. It may also be beneficial to have a PSW attend group therapy sessions to provide expert knowledge that some participants in this study requested more of while indirectly providing other positive benefits associated with PSW, including the opportunity of model learning, increased sense of empowerment, self-efficacy, and hope (73–77).

Furthermore, some participants stated the need for ongoing support. Future research should extend the number of sessions, as more than eight sessions might be needed to convey and practice different emotion regulation strategies, including mindfulness. More time to practice the strategies may help implement the exercises into everyday life. Also, patients’ engagement could be higher as they had more time to get used to group therapy settings, and the hesitation to reveal personal information in the group setting may decrease. Another way to help implement exercises into everyday life is via digital technologies, e.g., an app and/or a daily reminder to practice the exercises and/or a digital diary. Lastly, the time to acquire data before and after the group therapy sessions should be reduced, if possible, so that patients feel less overwhelmed and more time can be allocated to the therapy itself.

## 5 Conclusion

In summary, the presented analysis addressed personal experiences with and personal changes resulting from the Feel-Good group treatment. Our data suggest that the group treatment was perceived as very helpful and resulted in numerous changes regarding overall well-being and one’s relation to emotions. The qualitative results fit well with the quantitative analysis (43) and offer additional important insight into change processes perceived by patients.

## Data Availability

The datasets presented in this article are not readily available because the participants did not agree to share the entire interview per se which is why the transcripts cannot be shared. However, the categories that have been formed may be shared on request. Requests to access the datasets should be directed to karolina.leopold@vivantes.de.
