# Supplementary material for: Subjective experiences of an acceptance and mindfulness-based group intervention (Feel-Good-Group) in young people with early psychosis
Source: Front Psychiatry. 2024 Oct 7;15:1369629. doi: 10.3389/fpsyt.2024.1369629 (PMC11492832; doi:10.3389/fpsyt.2024.1369629)
Supplement: Supplementary file 1 [file Table1.docx]

**Supplement**

**Subjective experiences of an Acceptance and Mindfulness-Based Group Intervention (Feel-Good-Group) in young people with early psychosis.**

This supplement contains supporting information for the manuscript mentioned above. Theme five is reported along with a table.

**Table S1:** Description of measures used in the study **1**

**Table S2:** Demographical and Clinical Characteristics between Subgroups **4**

**Text S1:** Theme 5: Wishes and suggestions to change/modify the intervention. **5**

**Table S2:** Patient quotations on wishes and suggestions to change/modify the intervention.  **6**

## Table S1: Description of measures used in the study

| **Name** | **Description** | **Length (range in minutes in this study)** |
| --- | --- | --- |
| ***Screening*** | | |
| Structured Clinical Interview for DSM-IV (SCID)^1^ | A semi-structured interview for making diagnoses according to the diagnostic criteria published in the Diagnostic and Statistical Manual of Mental Disorders (DSM-IV). | 60-150 |
| Mehrfachwahl-Wortschatzt_Intelligenztest (MWT-B)^2^ | Estimate of Verbal Intelligence | 5-15 |
| ***Primary outcome measures*** | | |
| Goal Attainment Scale (GAS)^3^ | is an interview that was used to help patients formulate at least two emotion-oriented goals to work towards with the help of the Feel-Good group. Specific emotional therapy goals would be identified, i.e., “I want to be less angry” would be explored and a more specific goal would be set, such as “I would like to find better strategies on how to deal with my anger. Now I tend to release it by screaming in public or hitting things, such as the  wall”). The scores ranged from “much less than expected” (-2) to “more than expected” (+2). At both post-intervention assessments, patients estimated whether their individual goals were achieved. | 60-80 |
| Positive and Negative Syndrome Scale (PANSS)^4^ | is a semi-structured interview used to assess psychotic symptom severity. The interview is divided into three scales and is scored using a 7-point Likert scale (PANSS positive scale (7 symptoms: range 7-49), PANSS negative scale (7 symptoms, range: 7-49) and PANSS general psychopathology scale (16 symptoms, range: 16-112). For this study, the total PANSS score was used as primary outcome. In addition, the subscales were used as secondary outcome variables | 40-60 |
| ***Secondary outcome measures*** | | |
| Calgary Depression Scale for Schizophrenia (CDSS)^5^ | a semi-structured interview utilized to assess depressive symptoms in people with schizophrenia. A total CDSS score was computed and used by adding all items together (range 0-21). | 20-40 |
| Role Functioning Scale (RFS)^6^ | measures the level of functioning in four different domains: working productivity, independent living and self-care, immediate social network relationships (family and friends), and extended social network relationships (other social contacts). There are five items, which were used to compute a mean score (range 12-48). Higher scores indicate higher social functioning. | 5-10 |
| Paranoia Checklist (PCL)^7^ | is an 18-item self-report questionnaire that examines the frequency, distress, and conviction of paranoid delusions/suspicious thoughts. Items are answered and rated using a 5-point Likert scale. Higher scores indicate more paranoid/suspicious thoughts present. All three subscale scores were utilized in this study. | 10-15 |
| Psychotic Symptom Rating Scale (PSYRATS)^8^ | is made up of two different scales: delusions and hallucinations (PSYRATS-D and PSYRATS-H, respectively). Both scales are based on a semi-structured interview that assess different aspects of the psychotic symptoms, including the amount and duration of preoccupation, conviction, disruption of daily life, and amount and intensity of distress.  PSYRATS-H is comprised of 11 items (range: 0-44), whereas PSYRATS-D is comprised of 6 items (range: 0-24). All items are answered on a 5-point Likert scale. | 10-20 |
| Peters et al. Delusions Inventory (PDI)^9^ | s a 21-item questionnaire split into four subscales: endorsed delusions, delusional beliefs, frequency of delusional beliefs and conviction of delusional beliefs. Participants are first asked whether they endorse a delusional belief and are then asked to rate the frequency, preoccupation, distress and conviction of that belief on a 7-point-Likert scale. A PDI Total score was computed by summing up the subscales (range 0 – 336). | 10-20 |
| ***Putative mediators (Emotion Regulation)*** | | |
| Beliefs about Stress Scale (BASS)^10^ | is a 19-item questionnaire exploring three dimensions of stress: negative stress beliefs, positive stress beliefs and controllability of stress (BASS-N: range [8-32]; BASS-P: range [3-12]and BASS-C: range [4-16], respectively). | 5-15 |
| Emotion Regulation Inventory (ERI)^11^ | assesses a patients’ ability to utilize strategies to regulate negative (ERI-NE) and positive (ERI-PE) emotions. There are 47 items that are answered and scored using a 5-point Likert scale (0 = “never applies” to 4= “always applies”). | 10-25 |
| Emotion Regulation Questionnaire (ERQ)^12^ | is a 10-item questionnaire designed to measure the tendency individuals have to regulate their own emotions in terms of Cognitive Reappraisal (ERQ-C) and Expressive Suppression (ERQ-S). All items are answered on a 7-point Likert-scale ranging from 1 (strongly disagree) to 7 (strongly agree) (range: 10-70). | 5-10 |
| Rosenberg Self-Esteem Scale (RSE)^13^ | is a 10-item scale that assesses global self-worth. It measures both positive and negative feelings about the self. All items are answered using a 4-point Likert scale that ranges from 1 (strongly disagree) to 4 (strongly agree) (range: 0-30) | 5-15 |
| Self-Compassion Scale (SCS)^14^ | is a 26-item questionnaire that measures six areas of self-compassion: self-kindness, self-judgement, common humanity, isolation, mindfulness and over-identification. The total SCS score is computed by summing up all the mean values of the subscales and calculating the total mean, with higher scores indicating high self-compassion (range: 1-5). | 10-25 |
| Emotion Regulation Skills Questionnaire (ERSQ)^15^ | is a self-report on emotion regulation skills with 27 items rated on a 5-point Likert scale (range 0-108). In the present study, the total score is used. | 10-25 |

References:

1. First MB, Williams JBW, Karg RS, Spitzer RL. Structured Clinical Interview for DSM-5, Research Version; SCID-5-RV. Arlington, VA: American Psychiatric Association; 2015.
2. Lehrl S. Mehrfachwahl-Wortschatz-Intelligenztest. Balingen: Spitta Verlag; 2005.
3. Kiresuk TJ, Sherman RE. Goal attainment scaling: A general method for evaluating comprehensive community mental health programs. Community Ment Health J. 1968 Dec;4(6):443–53.
4. Kay SR, Fiszbein A, Opler LA. The positive and negative syndrome scale (PANSS) for schizophrenia. Schizophr Bull. 1987;13(2):261–76.
5. Müller MJ, Marx-Dannigkeit P, Schlösser R, Wetzel H, Addington D, Benkert O. The Calgary Depression Rating Scale for Schizophrenia: development and interrater reliability of a German version (CDSS-G). J Psychiatr Res. 1999 Oct;33(5):433–43.
6. Goodman SH, Sewell DR, Cooley EL, Leavitt N. Assessing levels of adaptive functioning: the Role Functioning Scale. Community Ment Health J. 1993 Apr;29(2):119–31.
7. Freeman D, Garety PA, Bebbington PE, Smith B, Rollinson R, Fowler D, et al. Psychological investigation of the structure of paranoia in a non-clinical population. Br J Psychiatry J Ment Sci. 2005 May;186:427–35.
8. Haddock G, McCarron J, Tarrier N, Faragher EB. Scales to measure dimensions of hallucinations and delusions: the psychotic symptom rating scales (PSYRATS). Psychol Med. 1999 Jul;29(4):879–89.
9. Peters E, Joseph S, Day S, Garety P. Measuring delusional ideation: the 21-item Peters et al. Delusions Inventory (PDI). Schizophr Bull. 2004;30(4):1005–22.
10. Laferton J, Stenzel N, Fischer S. The Beliefs About Stress Scale (BASS): Development, Reliability, and Validity. Int J Stress Manag. 2016 Sep 26;25.
11. König D. Die Regulation von negativen und positiven Emotionen. Entwicklung des Emotionsregulations-Inventars und Vergleich von Migränikerinnen und Kontrollpersonen [Internet]. [Austria]: University of Vienna; 2011. Available from: <https://dk.akis.at/emotion_regulation_inventory.html>
12. Gross JJ, John OP. Individual differences in two emotion regulation processes: implications for affect, relationships, and well-being. J Pers Soc Psychol. 2003 Aug;85(2):348–62.
13. Rosenberg M. Society and the Adolescent Self-Image [Internet]. Society and the Adolescent Self-Image. Princeton University Press; 2015 [cited 2022 Mar 7]. Available from: <https://www.degruyter.com/document/doi/10.1515/9781400876136/html>
14. Neff KD. The Development and Validation of a Scale to Measure Self-Compassion. Self Identity. 2003 Jul 1;2(3):223–50.
15. Grant M, Salsman NL, Berking M. The assessment of successful emotion regulation skills use: Development and validation of an English version of the Emotion Regulation Skills Questionnaire. PloS One. 2018;13(10):e0205095.

## Table S2: Demographical and Clinical Characteristics between Subgroups

| **Sociodemographic and Clinical Baseline characteristics** | Interview Sample *(n=10)* | | Non-Interview Sample *(n=11)* | | Test Statistic | | |
| --- | --- | --- | --- | --- | --- | --- | --- |
|  | M / N | SD / % | M / N | SD / % / (Range) | *t (20)*/ χ^2^ | *p* | Cohen’s *d* /Φ |
| Age (years) | 24.20 | 6.84 | 23.09 | 3.86 | -.45 | .66 | 5.48 |
| Gender (Female) | 4 | 40.0 | 5 | 45.5 | .06 | .80 | -.06 |
| Marital Status (single): | 10 | 100 | 11 | 100 |  |  |  |
| Education (years) | 13.06 | 2.13 | 13.4 | 2.47 | -.12 | .91 | 2.29 |
| Number of Psychotic Episodes: |  |  |  |  | 3.96 | .41 | .43 |
| 1st Episode | 5 | 50 | 3 | 27.3 |  |  |  |
| 2 Episodes | 3 | 30 | 5 | 45.5 |  |  |  |
| 3+ Episodes | 2 | 20 | 3 | 27.3 |  |  |  |
| Family History of Mental Illness^a^: | 9 | 90 | 10 | 91 | .01 | .94 | -.02 |
| Schizophrenia | 1 | 10 | 0 | 0 |  |  |  |
| Bipolar Disorder | 0 | 0 | 2 | 18.2 |  |  |  |
| Depression | 4 | 40 | 7 | 63.6 |  |  |  |
| Alcohol Dependency | 3 | 30 | 0 | 0 |  |  |  |
| Drug Dependency | 0 | 0 | 1 | 9.1 |  |  |  |
| Anxiety | 1 | 10 | 1 | 9.1 |  |  |  |
| PANSS Total Score^b^* | 70.80 | 20.11 | 66.27 | 16.78 | .56 | .58 | 18.43 |
| PANSS Total Scoref* | 49.10 | 12.35 | 52.27 | 17.07 | -.23 | .83 | 9.50 |
| RFS Total Score^b^* | 40.40 | 10.76 | 39.45 | 8.20 | .49 | .63 | 15.02 |
| RFS Total Scoref* | 41.70 | 7.66 | 44.64 | 10.28 | .75 | .47 | 9.13 |

*Notes:* M = Mean; N= Number; SD = Standard deviation; PANSS = Positive and Negative Syndrome Scale Total Score; PANSS Total Score = PANSS Total Score at baseline; PANSS Total Score = PANSS Total Score at Follow-up (16 weeks); RFS Total Score = Role Functioning Scale; RFS^b^= Role Functioning Score at baseline; RFS^f^ = Role Functioning Score at Follow-Up (16 weeks).

## Text S2: Theme 5: Wishes and suggestions to change/modify the intervention.

Participants were asked about their wishes and suggestions for improving or modifying the Feel-Good intervention. Numerous points were raised regarding helpful aspects and things that should be changed or added to improve the Feel-Good intervention.

Patient engagement

Two patients desired to boost patient engagement and communication among the participants within the group therapy sessions. P10 proposed encouraging more dialogue between patients (see Table S2). Furthermore, P9 suggested creating more interactive sessions, as they became tired in therapy sessions, making it harder to stay engaged (see Table S2).

Education

Two patients spoke about how the Feel-Good intervention should allow for a more in-depth exploration of why negative emotions arise (P3 and P8). For both patients, there was a lack of exploring the origins of negative emotions in example cases or one’s personal life (see Table S2). Two other patients (P2 and P5) spoke on how more example cases should be incorporated into the group therapy sessions to practice and improve implementing strategies to cope with distressing emotions (see Table S2). Also, P10 expressed a desire to include diagnostic comorbidities into the educational aspect of emotion regulation strategies, as they believed that the techniques learned in the Feel-Good group would have helped beyond the psychotic symptoms (see Table S2).

Mindfulness // Transition into everyday life

P6 revealed that the strategies learned in the group apply to everyday life. However, the strategies are not implemented automatically (see Table S2). Furthermore, P1 found mindfulness to be a very helpful concept and maintained that it should remain in the Feel Group intervention (see Table S2).

Therapy setting

Two patients (P5 and P10) suggested that the Feel-Good group should be longer than eight sessions or that one should be allowed to attend the Group intervention more than once to grasp the conveyed content better and help transition the strategies learned into everyday life (see Table S2). Furthermore, P10 also conveyed that they would rather partake in the Feel-Good group after their hospital stay due to the sedation they experienced because of the medication (see Table S2).

## Table S2: Patient quotations regarding wishes and suggestions to change/modify the intervention.

| **Participant** | **Quotation** |
| --- | --- |
| ***Patient engagement*** | |
| P10 | P: „Yeah, so, I would find it nice if the dialogue would be encouraged a bit more.  I: “The Dialogue between the Participants?”  P: “Between the participants, yes. Because that was done less. There is always the dialogue between the therapists and the participants, but I actually think amongst us [participants] it would be nice if we could do something more there”. |
| P9 | “Hmm, no, well, all I know is that I think I got a little tired sometimes from all the sitting. Perhaps the group could have been made a little more active. But, I don’t know how, maybe by standing up or something or including role-playing games […].” |
| ***Education*** | |
| P3 | “That you can describe these negative feelings better and find their reason [why the negative feelings are there] and discuss them. Why is that? And why do you get such negative feelings? If you find the reasons, you will probably be better at looking for a solution and can help yourself so that you can cope with this negative feeling.” |
| P8 | “Yes, I actually thought it would be a little deeper. When I think about, well psychological conversations, somehow I would have thought that they would be even more profound, go back to the origins. And somehow I was wondering a little bit whether it all goes back to the origins. There, where it actually comes from. Sometimes I missed the ‘why.’” |
| P2 | “So a little more specific about what we can do when such feelings arise, perhaps even more suggestions for solutions from the therapists” |
| P5 | P: “ Maybe one could somehow describe different situations. And then, so [situations] that are deep, that you make up and then somehow you try to find the best solutions […]”  I: “So, that’s a little more specific…”  P: “Yes, so that you case studies and then you collect different ideas about how things could have been handled better. Or how to deal with this feeling better in this situation.” |
| P10 | “I was hoping that it would be more about Obsessive Compulsory Disorder. […] because it was more focused on psychosis. I was hoping that it would be a little more about […] I would have been happy then because I believe that behavioural therapy could really help me. […] That would have just been nice. That would have been something, I think, that could have been integrated well. […] That would have been cool, if that could have been covered as well. I personally would have been interested in that, as it also has a lot do with emotions, I believe.” |
| ***Mindfulness*** | |
| P6 | “Yes, so sometimes I find it difficult, that you don’t even think about [utilizing the strategies]. The things you learned in the group that you can actually implement in everyday life. Or sometimes, I forget that I could implement it now, in everyday situations […]” |
| P1 | “Definitely keep the Mindfulness exercises, it is a good option, and, at the same time, there is good training for that”. |
| ***Therapy setting*** | |
| P5 | “Maybe it could have been a bit longer. I don’t think it was that many hours, but it was still quite pleasant, to explain the group’s intent” |
| P10 | “If it would go over a longer period of time, if one could say, that it somehow occurs during the inpatient stay and afterwards, that it does not only occur during the acute bad phase. I think, I would have profited more from the group.”  “Probably more therapy would help. To continue to deepen it further or make it more regular. […]. If it went on for longer or if you could take part several times, I would think that would be good”. |
| P10 | “ I would almost rather do the group now. […], because I was heavily sedated through the medication and sometimes fell asleep during the group”. |
